# A survey of radiation dose to patients and operators during radiofrequency ablation using computed tomography

**DOI:** 10.2349/biij.6.1.e2

**Published:** 2010-01-01

**Authors:** A Saidatul, CA Azlan, MSA Megat Amin, BJJ Abdullah, KH Ng

**Affiliations:** 1 Biomedical Electronics Engineering Program, University Malaysia Perlis, Arau, Malaysia; 2 Department of Biomedical Imaging, University of Malaya, Kuala Lumpur, Malaysia

**Keywords:** radiofrequency ablation, Gafchromic film, thermoluminescent dosimeter, computed tomography fluoroscopy

## Abstract

Computed tomography (CT) fluoroscopy is able to give real time images to a physician undertaking minimally invasive procedures such as biopsies, percutaneous drainage, and radio frequency ablation (RFA). Both operators executing the procedure and patients too, are thus at risk of radiation exposure during a CT fluoroscopy.

This study focuses on the radiation exposure present during a series of radio frequency ablation (RFA) procedures, and used Gafchromic film (Type XR-QA; International Specialty Products, USA) and thermoluminescent dosimeters (TLD-100H; Bicron, USA) to measure the radiation received by patients undergoing treatment, and also operators subject to scatter radiation.

The voltage was held constant at 120 kVp and the current 70mA, with 5mm thickness. The duration of irradiation was between 150-638 seconds.

Ultimately, from a sample of 30 liver that have undergone RFA, the study revealed that the operator received the highest dose at the hands, which was followed by the eyes and thyroid, while secondary staff dosage was moderately uniform across all parts of the body that were measured.

## INTRODUCTION

While the field of view (FOV) is concentrated on a more limited area in CT fluoroscopy, conventional CT uses 150-170 mA, and CT fluoroscopy tube current is invariably reduced to 50 mA. Other dosimetry practices are similar to the usual spiral CT scanning, along with tube voltage (kVp) being at similar levels.

These real-time 2-D images are constantly updated while the x-ray tube rotates around the subject. Dynamic axial imaging is provided, with real-time observation of image reconstruction possible. Given this advantage, it is an invaluable procedure for monitoring the passage of a biopsy needle, or the tube during drainage.

A notable divergence in the two techniques is that CT fluoroscopy is an interactive process, and as such requires the presence of an operator at the location of the gantry. This necessarily invokes exposure to scatter radiation, while conventional CT scanning allows the operator to control the process remotely.

A non-surgical procedure, such as radio frequency ablation (RFA), is a localised treatment that offers the patient freedom from side effects without interfering with their general health, and a rapid return to normal activity.

RFA uses the device of radiofrequency energy to which is inserted into target tissue (invariably a tumour) via the tip of a needle through the patient’s skin. On contact with the target tissue heat is produced, which kills the target tissue while sparing tissue that is healthy. In the event of the tumour causing bone matter to decay; this will not regenerate however, the dead tissue of a tumour will reduce in size.

## MATERIALS AND METHODS

The University of Malaya Medical Centre provided the opportunity to gather the data for this study from thirty RFA liver treatments. The patients underwent sedation and the staff involved included a radiologist and anaesthetist, medical officers, a radiographer, along with nurses and support staff. The same radiologist and support staff participated in all 30 monitored treatments. Tube voltage was constant at 120 kVp, tube current at 70 mA and slice thickness was 5mm.

### Patient Dose

**Figure 1:**
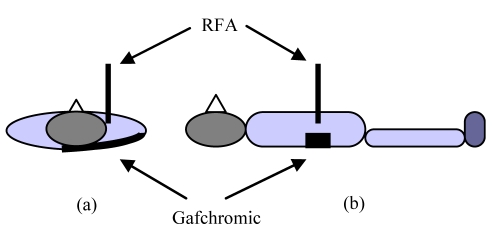
Diagram showing how Gafchromic film is positioned at the patient’s back.

The use of film in dosimetry provides precise information in respect of the location of the dose to the skin, and with calibration of the film, it also allows for quantitative measurement of the dose. To this end, Gafchromic® type XR-QA film (5" x 6") was placed at the rear of the patient (Fig. 1), and the radiation received during treatment was measured.

Afterward, the film was converted to a digital format using a Mikrotek ScanMaker 4800 flatbed scanner (Carson, USA) with ScanWizard5 Version 7 software. Measuring the extent of the blackened area on the digital format allowed identification of the radiation dose received, and was made possible by Adobe Photoshop version 6.0 (Adobe Systems, California, USA).

### Operator Dose

**Figure 2:**
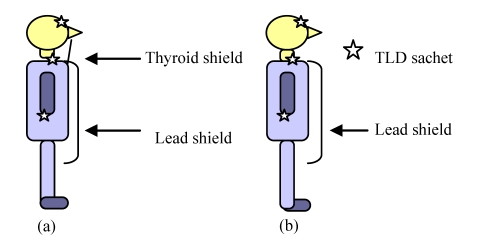
The TLD sachets were taped at the forehead, thyroid and middle finger of the radiologist and supporting staff (a) Radiologist – the TLD sachet was taped under the thyroid shield (b) Supporting staff – TLD sachet was taped at the neck. Supporting staff did not wear thyroid shield.

Thermo luminescent Dosimeters (TLD’s) were used to measure the scatter radiation received by the operator and other staff. Each individual had a sachet containing three TLD chips attached at the forehead, thyroid and middle finger (Fig. 2), and the radiologist alone wore a thyroid shield. A TLD reader (Harshaw 3500, Bicron, Ohio) then determined the average of these.

## RESULT AND DISCUSSION

### Patient Dose

**Figure 3:**
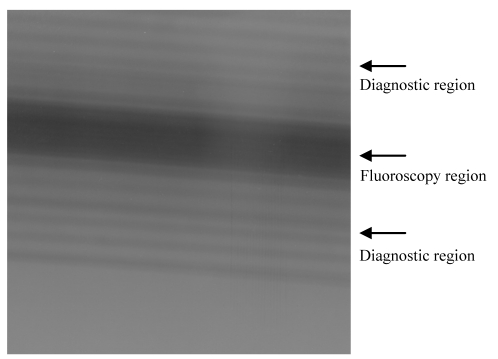
The gafchromic film image after being exposed.

Radiation exposure during an RFA procedure that causes blackening on the Gafchromic film is shown in Fig. 3. Due to the fact that the x-ray tube has a constant orbit around the body of the patient, the darker stripes signifying higher doses of radiation are found in the same central location. Other alternating light and dark stripes occur below and above the dark band at the centre due to scout and helical scanning that is used to locate the target area prior to the insertion of the radio frequency probe into the liver.

The radiation dose received at the entry point on the skin is provided as a function of time in Table 1, which shows that the dosage received increases with time. While the highest dosage received was 326±2.12 µGy for the duration 9<t<10 minutes, 326 µGy is not considered particularly harmful to a patient.

**Table 1:** Entrance dose in relation to exposure time.

**Exposure time (min)**	**No. of cases, n**	**Entrance Skin Dose (mGy)**
2 < t < 3	3	93 ± 7.3
3 < t < 4	3	104 ± 3.8
4 < t < 5	4	148 ± 5.0
5 < t < 6	4	163 ± 2.2
6 < t < 7	4	194 ± 4.6
7 < t < 8	6	207 ± 2.1
8 < t < 9	3	286 ± 3.1
9 < t < 10	3	326 ± 2.1

The World Health Organization (WHO) holds that skin erythema and temporary epilation of the skin will occur at dosage levels of 2 and 3 µGy, yet the basis for this study was conducted using a mere 32 µGy/min [2]. Ordinarily, CT fluoroscopy uses 120 kVp tube voltage and 70 mA tuber current for a typical RFA liver procedure, and with an exposure of approximately 90 minutes, will invariably cause skin erythema and temporary epilation of the skin to occur.

### Operator Dose

Given that the dosage received will increase as time increases, the scatter radiation received by the radiologist and support staff conform to this presumption (Tables 2 and 3).

**Table 2:** Dose received by the radiologist in relation to duration of procedures.

**Duration (min)**	**No.of cases, n**	**Scattered Radiation Dose(Radiologist (µGy))**		
		**Eye**	**Thyroid**	**Hand**
2 < t < 3	3	104 ± 3	29 ± 3	102 ± 2
3 < t < 4	3	113 ± 4	31 ± 6	108 ± 1
4 < t < 5	4	154 ± 11	36 ± 1	194 ± 4
5 < t < 6	4	160 ± 8	51 ± 4	213 ± 5
6 < t < 7	4	179 ± 11	53 ± 19	227 ± 4
7 < t < 8	6	184 ± 5	54 ± 2	273 ± 2
8 < t < 9	3	245 ± 5	67 ± 1	301 ± 28
9 < t < 10	3	260 ± 4	73 ± 7	354 ± 4

**Table 3:** Dose received by the supporting staff in relation to duration of procedures.

**Duration (min)**	**No.of cases, n**	**Scattered Radiation Dose(Supporting Staff (µGy))**		
		**Eye**	**Thyroid**	**Hand**
2 < t < 3	3	16 ± 2	15 ± 1	16 ± 1
3 < t < 4	3	21 ± 1	16 ± 5	18 ± 1
4 < t < 5	4	22 ± 4	17 ± 1	21 ± 1
5 < t < 6	4	24 ± 2	18 ± 1	20 ± 2
6 < t < 7	4	26 ± 3	19 ± 2	23 ± 1
7 < t < 8	6	31 ± 6	27 ± 3	32 ± 5
8 < t < 9	3	38 ± 2	32 ± 2	32 ± 1
9 < t < 10	3	47 ± 3	40 ± 1	37 ± 2

**Table 4:** Dose rate received by operators (radiologist and supporting staff) at eye, thyroid and skin of hand.

**Organ**	**Radiologist (μGy/min)**	**Supporting staff (μGy/min)**
Eye	31 ± 2	5 ± 2
Thyroid	9 ± 1	4 ± 1
Skin	37 ± 4	4 ± 1

The distance from the x-ray tube proved to be fundamental to the extent of radiation received.

Accordingly, the radiologist being the closest to the tube, received the greatest dose to the hand (354±4 µGy), and less radiation to the eye (260±4 µGy) and the thyroid (73±7 µGy). In order to insert the RF needle into the tumour, the radiologists hand is quite close to the gantry, and this provides sound basis of the results that emerged.

The rate of exposure also proved illuminating, as the radiologist recorded higher levels to the hand (37±4 µGy/min) and eye (31± µGy/min), yet only a mere 9±1 µGy/min at the thyroid.

Of course, this is basis for the contention that a thyroid shield will dramatically reduce exposure to radiation, with dose rates recorded at a reduction of 24-29% compared to the unprotected eye and skin.

As one would expect, the supporting staff underwent far lower levels of scatter radiation.

Here, the eye received the greatest dose (47±3 µGy ) with the thyroid (40±1 µGy), and lastly the skin (37±3 µGy). Due to the steep increase in distance away from the gantry, and the negligible difference in distance of the eye, thyroid and skin, the distribution of these results was limited.

No thyroid shield was worn by supporting staff, and the rate of dosage received also supported its use in order to reduce exposure to scatter radiation. For the eye, thyroid and hand respectively, the data showed rates of 5±2 µGy/min, 4±1 µGy/min, and 4±1 µGy/min.

## CONCLUSIONS

While careful calibration is required, the essential element to this study was the use of Gafchromic film Type XR-QA. With it, precise quantitative measurements are possible when measuring the distribution of radiation from CT fluoroscopy.

Due to the x-ray beam in CT fluoroscopy orbiting along the same channel, radiation is concentrated upon that area, the tissue surrounding which may be at risk. Conversely, the preliminary diagnostic scanning process did not record comparable results.

The variables that influence the radiation received by patients and incidence of scatter radiation to operators are specifically, the duration of the procedure, the distance away from the gantry, and the dosimetric parameters.

Clearly, radiologists and supporting staff need to be aware of the risks associated with exposure to scatter radiation, and adopt occupational health and safety practices in wearing lead aprons, gloves and a thyroid shield.
